# Supplementary material for: Gut microbiota impacts bone via Bacteroides vulgatus-valeric acid-related pathways
Source: Nat Commun. 2023 Oct 27;14:6853. doi: 10.1038/s41467-023-42005-y (PMC10611739; doi:10.1038/s41467-023-42005-y)
Supplement: Supplementary file 1 — Supplementary information [file 41467_2023_42005_MOESM1_ESM.pdf]

Content

Extended Data.. 2

Table S1. BMD and VA-associated bacterial species and covariates at various skeletal sites in the Chinese cohort...2

Table S2. BMD-associated SCFAs and covariates at various skeletal sites...............................7

Table S3. Characteristics of the SNPs used in MR analysis..8

Table S4. Characteristics of US post-menopausal white female cohort..................................10

Table S5. BMD-associated gut bacterial species and covariates at various skeletal sites in the US cohort..11

Table S6. Regression coefficients between *B. vulgatus* and varies sites of BMD.................. 13

Table S7. Exclusion criteria for Chinese study subjects..15

Figure S1. Associations of life-style factors/socioeconomic status with BMD/SCFA............ 16

Figure S2. QQ plots of GWAS..17

Figure S3. Effects of different concentrations of valeric acid on osteoclast-like cells and osteoblasts.. 18

Online resources..19

List of abbreviations..21

Extended Data

Table S1. BMD and VA-associated bacterial species and covariates at various skeletal sites in the Chinese cohort

Variables	L1-L4 BMD		UD-RU BMD		HTOT BMD		Valeric acid	
	β	*p*-value	β	*p*-value	β	*p*-value	β	*p*-value
**YSM**	**-0.028**	**< 0.001**	**-0.016**	**< 0.001**	**-0.016**	**0.008**	-0.024	0.336
Age	-0.002	0.384	-0.001	0.136	-0.001	0.594	0.007	0.371
**BMI**	**0.014**	**< 0.001**	**0.006**	**< 0.001**	**0.015**	**< 0.001**	-0.013	0.117
**Exercise**	**0.025**	**0.001**	0.003	0.246	**0.014**	**0.015**	NA	NA
**Family annual income**	**0.023**	**0.017**	0	0.999	0.008	0.259	NA	NA
FSH	0	0.657	0	0.439	0	0.126	0	0.56
**Bacteroides_vulgatus**	**-0.027**	**0.032**	-0.004	0.369	-0.011	0.253	**-0.111**	**0.006**
Faecalibacterium_prausnitzii	0.003	0.789	0.002	0.564	0	0.972	-0.003	0.916
Bacteroides_uniformis	-0.002	0.867	-0.003	0.489	-0.004	0.629	0.023	0.5
Bacteroides_fragilis	0	0.986	-0.001	0.853	0.011	0.402	0.06	0.267

Escherichia_coli	0	0.84	0	0.84	0	0.784	0	NA
Bacteroides_stercoris	0	0.975	0.002	0.428	0.006	0.198	-0.01	0.585
**Bacteroides_ovatus**	-0.003	0.85	**0.011**	**0.046**	0.005	0.639	-0.054	0.225
Bacteroides_massiliensis	-0.001	0.896	0.006	0.088	-0.008	0.259	0.028	0.325
Eubacterium_eligens_cag72	-0.002	0.697	-0.001	0.464	0.001	0.744	0.005	0.719
Firmicutes_bacterium_cag65	-0.001	0.803	-0.001	0.707	0.001	0.798	0.021	0.229
Eubacterium_rectale	0.005	0.586	0.005	0.15	0.006	0.381	-0.023	0.409
Phascolarctobacterium_sp_cag2	-0.001	0.801	-0.001	0.57	0	0.916	-0.004	0.759
Clostridium_sp_cag7	0.007	0.359	0.005	0.059	0.009	0.104	-0.041	0.087
Bacteroides_coprocola	0.007	0.403	0.002	0.624	0.008	0.185	-0.018	0.503
Roseburia_sp_cag18	-0.001	0.787	0	0.976	0	0.921	-0.013	0.421
**Alistipes_putredinis**	0.001	0.885	0	0.838	0.002	0.6	**0.054**	**＜ 0.00**1
Roseburia_inulinivorans	0.008	0.415	0.002	0.496	0.01	0.145	-0.005	0.856
Roseburia_intestinalis	-0.008	0.44	-0.002	0.701	-0.004	0.606	-0.029	0.403
**Bacteroides_caccae**	-0.003	0.776	-0.004	0.385	0	0.976	**0.089**	**0.009**
Subdoligranulum_variabile	-0.005	0.477	0.001	0.655	0.001	0.857	0.005	0.837

Bacteroides_xylanisolvens	0.003	0.839	-0.004	0.418	0.006	0.555	-0.021	0.625
**Bacteroides_thetaiotaomicron**	**0.027**	**0.021**	0.004	0.41	0.003	0.7	0.022	0.544
Bacteroides_finegoldii	-0.018	0.192	-0.008	0.116	-0.012	0.267	-0.038	0.389
Bacteroides_eggerthii	0.009	0.313	0.002	0.48	-0.001	0.907	-0.006	0.828
Parabacteroides_distasonis	-0.028	0.091	-0.004	0.558	-0.015	0.219	-0.012	0.812
Parabacteroides_merdae	0.004	0.615	-0.002	0.451	0.001	0.85	-0.013	0.618
**Bacteroides_cellulosilyticus**	0.014	0.202	0.005	0.292	0.013	0.129	**0.083**	**0.021**
**Bacteroides_intestinalis**	-0.015	0.278	-0.006	0.283	**-0.021**	**0.043**	**-0.121**	**0.006**
Hungatella_hathewayi	-0.003	0.843	-0.004	0.475	0.01	0.424	-0.03	0.55
**Butyrateproducing_bacterium_ss3/4**	**-0.024**	**0.04**	-0.005	0.258	**-0.022**	**0.009**	0.03	0.399
**Clostridium_bolteae**	**0.045**	**0.036**	0.014	0.072	0.023	0.152	-0.008	0.899
**Bacteroides_sp_9_1_42faa**	**0.033**	**0.031**	0.006	0.289	0.017	0.15	0.045	0.359
Roseburia_hominis	0.015	0.179	-0.001	0.781	0.003	0.755	0.017	0.63
Clostridium_sp_cag43	0.005	0.601	0.004	0.215	-0.001	0.847	0.009	0.753
uncultured_bacterium	0	NA	0	NA	0	NA	0	NA
Eubacterium_ventriosum	-0.003	0.801	-0.001	0.88	-0.002	0.798	0.022	0.52

Blautia_sp_cag37	-0.002	0.843	-0.005	0.122	-0.006	0.399	-0.003	0.913
Clostridium_clostridioforme	-0.028	0.418	-0.017	0.196	-0.025	0.326	0.134	0.216
Peptoclostridium_difficile	-0.049	0.095	0.003	0.761	0.012	0.575	0.056	0.543
Blautia_wexlerae	-0.003	0.865	-0.003	0.646	-0.003	0.841	-0.036	0.514
Ruminococcus_lactaris	0.01	0.475	0.005	0.361	0.008	0.421	0.053	0.225
Bilophila_wadsworthia	0	NA	0	NA	0	NA	0	NA
Flavonifractor_plautii	-0.002	0.903	-0.003	0.491	-0.012	0.186	-0.066	0.091
Bacteroides_sp_3_1_33faa	0	NA	0	NA	0	NA	0	NA
Tyzzerella_nexilis	0.017	0.388	0.004	0.606	0.014	0.366	-0.072	0.253
Dorea_formicigenerans	0.018	0.535	-0.003	0.779	-0.021	0.353	-0.02	0.827
Lachnospiraceae_bacterium_a4	0	NA	0	NA	0	NA	0	NA

Note:

β - regression coefficient for the association between variable and human BMD/valeric acid variation; *p*-value - *p*-value of the regression coefficient.

Bolded species were the nominally significant ones associated with BMD/valeric acid (*p*-values < 0.05).

Abbreviations:

YSM - years since menopause; BMD - bone mineral density; L1-L4 - lumbar spine; HTOT - left total hip; UD-RU - ultra-distal radius and ulna; FN - femoral neck; BMI - body mass index; FSH - follicle stimulating hormone.

Table S2. BMD-associated SCFAs and covariates at various skeletal sites

Variables	L1-L4 BMD		HTOT BMD		UD-RU BMD	
	β	*p*-value	β	*p*-value	β	*p*-value
Intercept	0.883	< 0.001	0.646	< 0.001	0.334	< 0.001
Caproic acid	-0.001	0.982	-0.014	0.416	-0.001	0.871
Isovaleric acid	-0.014	0.325	0.004	0.700	-0.002	0.724
Butyric acid	-0.037	0.213	-0.024	0.266	-0.013	0.233
Acetic acid	-0.005	0.686	-0.001	0.906	-0.001	0.884
Isobutyric acid	-0.010	0.510	-0.007	0.505	-0.001	0.871
**Valeric acid**	0.044	**0.017**	0.024	0.073	0.009	0.196
**YSM**	-0.024	**0.001**	-0.015	**0.008**	-0.016	**< 0.001**
Age	-0.003	0.245	-0.001	0.549	-0.001	0.157
**BMI**	0.015	**< 0.001**	0.016	**< 0.001**	0.007	**< 0.001**
**Exercise**	0.024	**0.002**	0.013	**0.022**	0.003	0.249
**Family annual income**	0.022	**0.020**	0.007	0.305	-0.001	0.782
FSH	< -0.001	0.625	< -0.001	0.139	< -0.001	0.333

Note:

β - regression coefficient for the association between variable and human BMD variation; *p*-value - *p*-value of the regression coefficient.

Bolded contents were the nominally significant ones associated with BMD (*p*-values < 0.05).

Abbreviations:

SCFAs - short chain fatty acids; YSM - years since menopause; BMD - bone mineral density; L1-L4 - lumbar spine; HTOT - left total hip; UD-RU - ultra-distal radius and ulna; BMI - body mass index; FSH - follicle stimulating hormone.

Table S3. Characteristics of the SNPs used in MR analysis

SNP	CHR	Exposure (*B. vulgatus*)						Outcome (valeric acid)			
		effect_allele	β	*p*-value	se	R^2	F	effect_allele	β	*p*-value	se
rs688811	1	T	-1.288	3.95E-06	0.276	0.056	29.483	T	0.038	0.776	0.132
rs3219142	1	A	-1.019	3.63E-07	0.198	0.065	34.551	A	0	0.999	0.095
rs11682148	2	A	-1.089	6.40E-07	0.216	0.063	33.416	A	-0.060	0.555	0.101
rs77875633	2	G	-1.471	6.63E-07	0.292	0.062	32.851	G	0.194	0.165	0.139
rs36201997	4	A	-0.794	3.77E-06	0.170	0.058	30.601	A	-0.130	0.111	0.082
rs61870507	10	A	-0.510	2.77E-06	0.107	0.059	31.162	A	0.064	0.215	0.051
rs140807263	11	A	-1.146	3.70E-06	0.245	0.052	27.262	A	0.251	0.036	0.120
rs1077028	14	C	-0.401	1.34E-07	0.075	0.067	35.690	C	0.047	0.195	0.036
rs35903260	16	T	-0.316	8.10E-07	0.063	0.063	33.416	T	0.059	0.052	0.030
rs8049150	16	G	-1.069	4.27E-06	0.230	0.057	30.041	G	0.046	0.676	0.109
rs1452791	18	A	0.313	5.30E-06	0.068	0.054	28.370	A	0.053	0.105	0.032
rs4892102	18	G	-0.366	9.16E-06	0.082	0.053	27.815	G	0.092	0.016	0.038

rs17705279	19	G	-0.796	1.71E-06	0.164	0.055	28.926	G	0.094	0.231	0.078
rs6516034	20	A	0.351	4.25E-06	0.075	0.054	28.370	A	-0.045	0.214	0.036
rs2051388	21	G	0.386	6.46E-06	0.085	0.058	30.601	G	-0.041	0.316	0.040

Note:

Genome-wide association study (GWAS) was performed to identify *B. vulgatus*/valeric acid related-SNPs. *P*-values are the summary statistics results of the GWAS.

Table S4. Characteristics of US post-menopausal white female cohort

Phenotypes	Max	Min	Mean	Standard Deviation
Age (years)	80.52	60.12	66.98	5.65
BMI (kg/m^2)	49.99	17.47	27.84	8.50
L1-L4 BMD (g/cm^2)	1.53	0.63	0.92	0.17
HTOT BMD (g/cm^2)	1.21	0.44	0.80	0.12
FN BMD (g/cm^2)	0.93	0.43	0.67	0.11
UD-R BMD (g/cm^2)	0.52	0.21	0.37	0.06
UD-U BMD (g/cm^2)	0.39	0.14	0.28	0.05
	**Yes**		**No**	
Alcohol drinking	47/59 (79.7%)		12/59 (20.3%)	
Smoking	23/59 (39.0%)		36/59 (61.0%)	
Regular exercise	44/59 (74.6%)		15/59 (25.4%)	

Abbreviations:

BMI - body mass index; BMD - bone mineral density; L1-L4 - lumbar spine; HTOT - left total hip; FN - femoral neck; UD-R - ultra-distal radius; UD-U - ultra-distal ulna.

Table S5. BMD-associated gut bacterial species and covariates at various skeletal sites in the US cohort

Variables	L1-L4 BMD		HTOT BMD		FN BMD	
	**β**	***p*-value**	**β**	***p*-value**	**β**	***p*-value**
**Exercise**	0.121	0.097	0.087	**0.045**	0.084	0.022
**Fracture**	-0.030	0.517	-0.086	**0.003**	-0.090	0.000
Age	0.006	0.288	0.004	0.294	0.006	0.040
BMI	0.005	0.214	0.004	0.078	0.002	0.361
Alistipes_shahii	0.001	0.860	0.000	0.962	-0.005	0.179
Bacteroides_thetaiotaomicron	0.006	0.510	0.002	0.725	-0.003	0.548
Alistipes_onderdonkii	-0.005	0.421	-0.001	0.742	-0.002	0.502
Odoribacter_splanchnicus	0.009	0.224	0.002	0.704	0.001	0.765
Bacteroides_caccae	0.002	0.828	0.005	0.251	0.008	0.027
Parabacteroides_distasonis	0.001	0.831	0.006	0.153	0.004	0.279
Parabacteroides_merdae	-0.006	0.520	-0.002	0.646	0.001	0.885
Bacteroides_ovatus	0.008	0.444	0.006	0.348	0.002	0.641
Bacteroides_stercoris	-0.002	0.816	0.001	0.852	0.003	0.342
**Bacteroides_vulgatus**	-0.002	0.862	**-0.018**	**0.029**	-0.012	0.084
Alistipes_putredinis	-0.012	0.103	0.001	0.879	0.002	0.582
Lachnospiraceae_bacterium_7_1	-0.026	0.300	-0.026	0.075	-0.016	0.192
Roseburia_intestinalis	0.005	0.671	0.013	0.062	0.013	0.026
Dorea_longicatena	0.013	0.209	0.008	0.210	0.006	0.209
Clostridium_bolteae	0.004	0.622	0.008	0.118	0.002	0.617
Roseburia_inulinivorans	0.002	0.879	0.005	0.488	0.005	0.408
**Eubacterium_eligens**	-0.013	0.100	**-0.010**	**0.034**	-0.010	0.013
**Roseburia_hominis**	-0.012	0.293	**-0.018**	**0.014**	-0.014	0.017
Eubacterium_siraeum	0.006	0.408	-0.007	0.113	-0.007	0.036

**Ruminococcus_torques**	0.003	0.863	**0.021**	**0.043**	0.020	0.022
Eubacterium_rectale	0.012	0.176	0.000	0.996	-0.001	0.779
Oscillibacter_unclassified	0.011	0.500	-0.002	0.821	-0.005	0.496
Faecalibacterium_prausnitzii	-0.004	0.786	0.009	0.281	0.008	0.242

Note:

β - regression coefficient for the association between variable and human BMD variation; *p*-value - *p*-value of the regression coefficient.

Bolded species were the nominally significant ones associated with BMD (*p*-values < 0.05).

Abbreviations:

BMD - bone mineral density; L1-L4 - lumbar spine; HTOT - left total hip; FN - femoral neck; BMI - body mass index.

Table S6. Regression coefficients between *B. vulgatus* and varies sites of BMD

Phenotype	Chinese cohort			the US cohort		
	**β**	***p*-value**	***q*-value**	**β**	***p*-value**	***q*-value**
Spine (L1-L4) BMD	-0.027	0.032	0.176	-0.002	0.862	0.862
L1 BMD	-0.027	0.027	0.176	-	-	
L2 BMD	-0.025	0.057	0.200	-	-	
L3 BMD	-0.031	0.024	0.176	-	-	
L4 BMD	-0.029	0.042	0.176	-	-	
UD-R BMD	-0.004	0.409	0.505	-0.007	0.115	0.244
UD-U BMD	-0.004	0.493	0.545	-0.003	0.411	0.457
RTOT BMD	-0.006	0.212	0.378	-0.008	0.147	0.244
UD-RU BMD	-0.004	0.369	0.484	-	-	
R-33 BMD	-0.013	0.039	0.176	-	-	
U-33 BMD	-0.005	0.520	0.546	-	-	
RU-33 BMD	-0.009	0.153	0.378	-	-	
UTOT BMD	-0.003	0.598	0.598	-	-	
RUTOT BMD	-0.005	0.328	0.459	-	-	
R-mid BMD	-	-		-0.007	0.171	0.244
R-13 BMD	-	-		-0.006	0.339	0.424
HTOT BMD	-0.011	0.253	0.409	-0.018	0.029	0.244
FN BMD	-0.012	0.216	0.378	-0.012	0.084	0.244
FN (upper) BMD	-0.012	0.200	0.378	-	-	
FN (lower) BMD	-0.011	0.279	0.419	-	-	
Hip (wards) BMD	-0.019	0.073	0.219	-	-	
Hip (troch) BMD	-0.011	0.198	0.378	-	-	
Hip (FS) BMD	-0.009	0.449	0.524	-	-	
WB TOT	-	-		-0.014	0.133	0.244

WB (sub) TOT	-	-	-0.015	0.085	0.244

Note:

Linear regression analysis was performed to identify the correlation between *B. vulgatus* and varies sites of BMD. False discovery rate was calculated for multiple testing correction.

β - regression coefficients of the correlations; *p*-value - *p*-value of the correlation coefficient; *q*-value - false discovery rate of the *p*-values.

Table S7. Exclusion criteria for Chinese study subjects

Number	Exclusion criteria
1	Used antibiotics, oestrogens, anticonvulsant or proton pump inhibitor medications in the past three months;
2	Underwent hysterectomy or bilateral ovariectomy;
3	Serious residuals from cerebral vascular disease;
4	Diabetes mellitus, except for easily controlled, non-insulin dependent diabetes mellitus;
5	Chronic renal disease manifest by serum creatinine > 1.9 mg/dL;
6	Chronic liver diseases;
7	Significant chronic lung disease;
8	Alcohol abuse as defined by those who drink alcohol regularly and cannot control themselves and become intoxicated at least once a week.
9	Corticosteroid therapy at pharmacologic levels currently, or for more than 6 months duration at any time;
10	Treatment with anticonvulsant therapy currently, or for more than 6 months duration at any time;
11	Evidence of other metabolic diseases or inherited bone diseases such as hyper- or hypoparathyroidism, Paget's disease, osteomalacia, osteogenesis imperfecta, or others;
12	Rheumatoid arthritis, except for minor cases that involve only hand joint and wrist;
13	Recent major gastrointestinal disease (within the past year) such as celiac disease, post-gastrectomy, Crohn's disease, ulcerative colitis;
14	Any other disease, treatment (e.g., bisphosphonates), or condition that would be an apparent non-genetic factor underlying the variation of BMD.

Figure S1. Associations of life-style factors/socioeconomic status with BMD/SCFA.

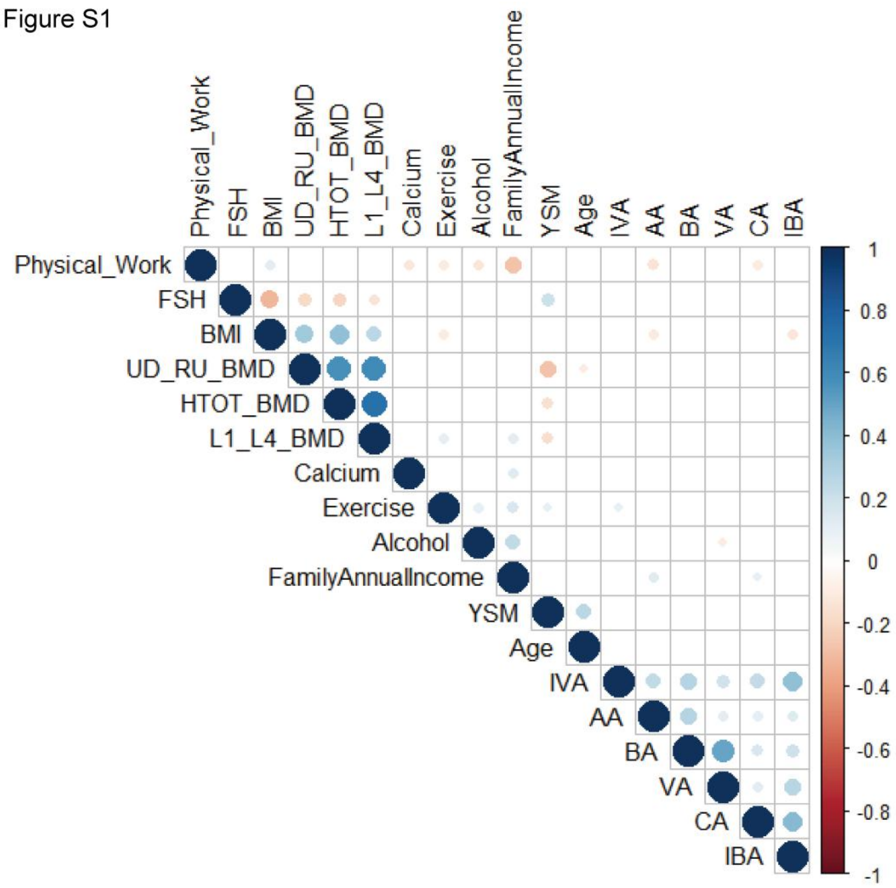

Note:

Colors (blue/red) and their intensity correspond to direction and strength of the correlations. Negative correlations are in red while positive correlations in blue. The darker the color, the stronger the correlation. The circle size corresponds to statistical significance (*p*-value), the larger, the more significant. Only the significantly associated factors with *p*-values < 0.05 are shown in the figure.

Abbreviations:

FSH - follicle stimulating hormone; BMI - body mass index; UD-RU - ultra-distal radius and ulna; BMD - bone mineral density; L1-L4 - lumbar spine; HTOT - left total hip; YSM - years since menopause; IVA - isovaleric acid; AA - acetic acid; BA - butyric acid; VA - valeric acid; CA - caproic acid; IBA - isobutyric acid.

Figure S2. QQ plots of GWAS.

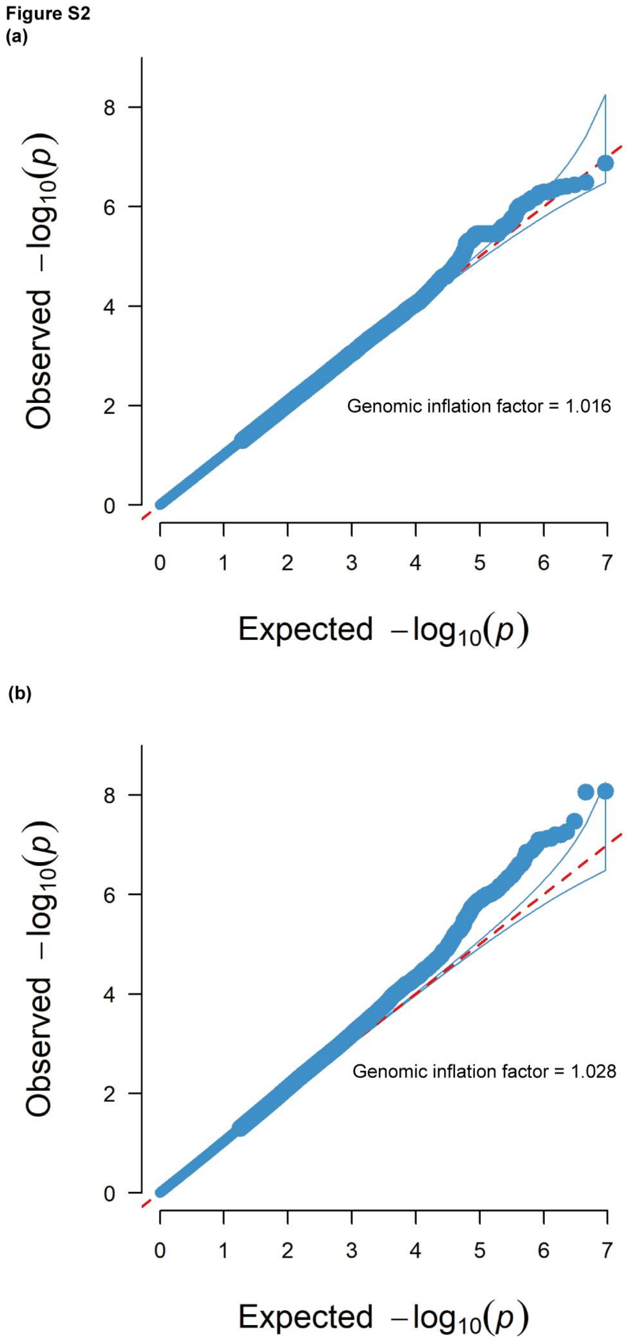

Note:

(a) QQ plot for GWAS data of *Bacteroides vulgatus*. (b) QQ plot for GWAS data of valeric acid.

Abbreviations:

GWAS - genome-wide association study.

Figure S3. Effects of different concentrations of valeric acid on osteoclast-like cells and osteoblasts

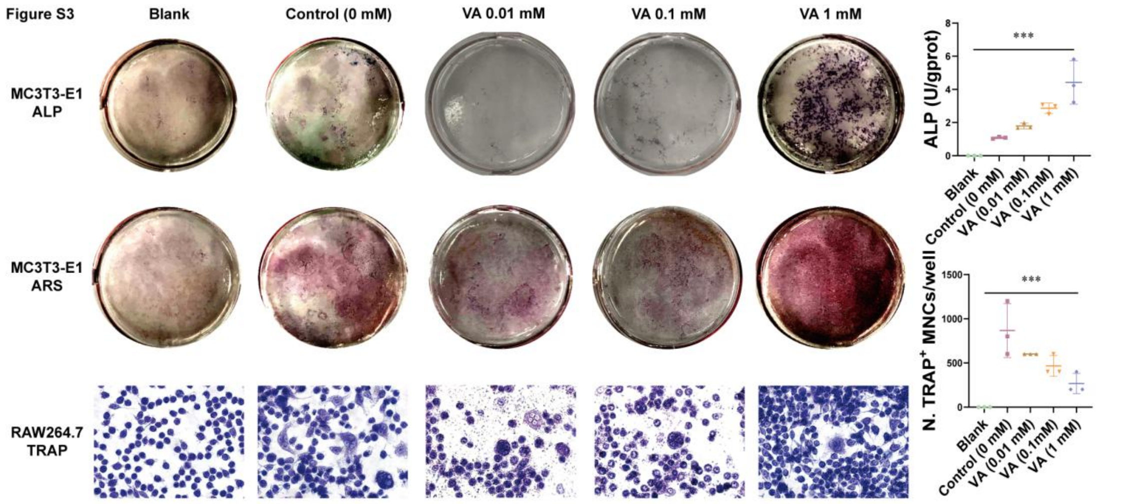

Note:

Effects of different concentrations of valeric acid (VA, 0 mM, 0.01 mM, 0.1 mM, and 1 mM) on osteoclast-like cells and osteoblasts. Alkaline phosphatase (ALP) staining and alizarin red S (ARS) staining were used to observe osteoblast differentiation and extracellular matrix mineralization, respectively. Tartrate-resistant acid phosphatase (TRAP) staining was used to observe osteoclast-like cells. Each experiment was repeated independently for three times.

MNC - multinucleated cells.

Online resources

Softwares	URL
Agilent Mass Hunter	https://www.agilent.com/en/promotions/masshunter-mass-spec
Bowtie2 v2.2.0	http://bowtie-bio.sourceforge.net/bowtie2/index.shtml
Burrows-Wheeler Aligner v0.7.17	https://sourceforge.net/projects/bio-bwa/
CD-HIT v4.6.1	http://weizhongli-lab.org/cd-hit/
Cutadapt v1.9	https://cutadapt.readthedocs.io/
DIAMOND v2.0.5	http://ab.inf.uni-tuebingen.de/software/diamond/
Fqtrim v0.94	http://ccb.jhu.edu/software/fqtrim/
Genome Analysis Toolkit 4	https://www.broadinstitute.org/gatk/
Kyoto Encyclopedia of Genes and Genomes	https://www.genome.jp/kegg/
MetaGeneMark v3.26	http://exon.gatech.edu/GeneMark/
PLINK 1.9	https://www.cog-genomics.org/plink/1.9/

R software v3.5.1	https://www.r-project.org/
SPAdes v3.10.0	http://cab.spbu.ru/software/spades/
Stata 14	https://www.stata.com/

List of abbreviations

Abbreviations	Full forms
β	regression coefficient
γ	correlation coefficient
Abs	absorbance
ALP	alkaline phosphatase
ARS	alizarin red S
BMD	bone mineral density
BMI	body mass index
B.V.	*Bacteroides vulgatus*
BV/TV	bone volume/tissue volume
CAMP	cationic antimicrobial peptide
CDS	coding sequences
CI	confidence interval
CLR	centered log ratio
CTX-I	C-telopeptide of type I collagen
CV	coefficient of variation
DMEM	Dulbecco’s Modified Eagle Medium
DXA	dual energy X-ray absorptiometry
E-value	expected value
EDTA	ethylene diamine tetraacetic acid
ELISA	enzyme-linked immune sorbent assay
FBS	fetal bovine serum
FFAR	free fatty acid receptor
FN	femoral neck

FSH	follicle stimulating hormone
GATK	genome analysis toolkit
GC-MS/MS	gas chromatography-tandem mass spectrometry
GDP	guanosine 5'-diphosphate
GM	gut microbiota
GTP	guanosine 5'-triphosphate
GWAS	genome-wide association study
HA	hydroxyapatite
HDAC	histone deacetylase
HE	hematoxylin-eosin
HTOT	left total hip
IHC-OCN	Immunohistochemistry-osteocalcin
IMP	inosine monophosphate
IVs	Instrumental variables
IVW	inverse-variance weighting
KEGG	Kyoto Encyclopedia of Genes and Genomes
KO	KEGG Orthology
L1-L4	lumbar spine
LD	linkage disequilibrium
microCT	micro-computed tomography
MaxLik	maximum likelihood estimation
MiRKAT	microbiome regression-based kernel association test
Md. Ar	mineralized area
MOST	China's Ministry of Science and Technology
MR	Mendelian randomization
NS	normal saline
N.OC^{+}/B.Pm	osteocalcin positive cells number per analyzed bone perimeter

N.TRAP$^+$/B.Pm	TRAP-stained osteoclast number per analyzed bone perimeter
TRAP$^+$MNCs	TRAP-positive multinucleated cells
OC	osteocalcin
one-way ANOVA	one-way analysis of variance
OP	osteoporosis
OVX	ovariectomized
p-	phosphorated
PINP	procollagen I N-terminal propeptide
PMOP	postmenopausal osteoporosis
PRPP	phosphoribosylpyrophosphate
qPCR	real-time quantitative polymerase chain reaction
QC	quality control
RANKL	receptor activator of nuclear factor-κB ligand
SCFAs	short chain fatty acids
SD	standard deviation
SNPs	single nucleotide polymorphisms
SPF	Specific pathogen free
Tb.N	trabecular number
Tb.Sp	trabecular separation
Tb.Th	trabecular thickness
TPM	transcripts per kilobase million
TRAP	tartrate-resistant acid phosphatase
UD-R	ultra-distal radius
UD-RU	ultra-distal radius and ulna
UD-U	ultra-distal ulna
VA	valeric acid
WGS	whole genome sequencing

WHO	world health organization
YSM	years since menopause
