# Supplementary material for: Gut microbiota impacts bone via Bacteroides vulgatus-valeric acid-related pathways
Source: Nat Commun. 2023 Oct 27;14:6853. doi: 10.1038/s41467-023-42005-y (PMC10611739; doi:10.1038/s41467-023-42005-y)
Supplement: Supplementary file 3 — Reporting Summary [file 41467_2023_42005_MOESM3_ESM.pdf]

nature portfolio

Corresponding author(s): Hong-Wen Deng, Jie Shen, Hong-Mei Xiao

Last updated by author(s): Oct 7, 2023

Reporting Summary

Nature Portfolio wishes to improve the reproducibility of the work that we publish. This form provides structure for consistency and transparency in reporting. For further information on Nature Portfolio policies, see our Editorial Policies and the Editorial Policy Checklist.

Statistics

For all statistical analyses, confirm that the following items are present in the figure legend, table legend, main text, or Methods section.

n/a	Confirmed	
☐	☒	The exact sample size (*n*) for each experimental group/condition, given as a discrete number and unit of measurement
☐	☒	A statement on whether measurements were taken from distinct samples or whether the same sample was measured repeatedly
☐	☒	The statistical test(s) used AND whether they are one- or two-sided<br>*Only common tests should be described solely by name; describe more complex techniques in the Methods section.*
☐	☒	A description of all covariates tested
☐	☒	A description of any assumptions or corrections, such as tests of normality and adjustment for multiple comparisons
☐	☒	A full description of the statistical parameters including central tendency (e.g. means) or other basic estimates (e.g. regression coefficient) AND variation (e.g. standard deviation) or associated estimates of uncertainty (e.g. confidence intervals)
☐	☒	For null hypothesis testing, the test statistic (e.g. *F*, *t*, *r*) with confidence intervals, effect sizes, degrees of freedom and *P* value noted<br>*Give P values as exact values whenever suitable.*
☒	☐	For Bayesian analysis, information on the choice of priors and Markov chain Monte Carlo settings
☐	☒	For hierarchical and complex designs, identification of the appropriate level for tests and full reporting of outcomes
☐	☒	Estimates of effect sizes (e.g. Cohen's *d*, Pearson's *r*), indicating how they were calculated

Our web collection on statistics for biologists contains articles on many of the points above.

Software and code

Policy information about availability of computer code

Data collection	Shotgun metagenomic sequencing data were collected by LC-Bio Technologies (Hangzhou) CO., LTD. (Hangzhou City, China, www.lc-bio.com). Short chain fatty acids data were collected by Wuhan Metware Biotechnology Co., Ltd (Wuhan City, China, www.metware.cn). Whole genome sequencing data were collected by BGI Genomics Co. Ltd (Shenzhen City, China; https://www.genomics.cn/). In vivo and in vitro experiments data were collected by Southern Medical University. All these information are also mentioned in the methods section of the manuscript. Softwares, including Agilent Mass Hunter, Burrows-Wheeler Aligner (v0.7.17), CD-HIT (v4.6.1), Cutadapt (v1.9), DIAMOND (v2.0.5), Fqtrim (v0.94), Genome Analysis Toolkit (v4), Kyoto Encyclopedia of Genes and Genomes, MetaGeneMark (v3.26), Bowtie2 (v2.2.0), and SPAdes (v3.10.0), were used during the data collection/analysis. All the information is mentioned in the supplementary information.
Data analysis	Statistical analyses were conducted using R (v3.5.1), Stata 14, PLINK 1.9. All these information are also mentioned in the methods section of the manuscript.

For manuscripts utilizing custom algorithms or software that are central to the research but not yet described in published literature, software must be made available to editors and reviewers. We strongly encourage code deposition in a community repository (e.g. GitHub). See the Nature Portfolio guidelines for submitting code & software for further information.

Data

Policy information about availability of data

All manuscripts must include a data availability statement. This statement should provide the following information, where applicable:

- Accession codes, unique identifiers, or web links for publicly available datasets
- A description of any restrictions on data availability
- For clinical datasets or third party data, please ensure that the statement adheres to our policy

The data that support the findings of this study have been deposited in public databases. The sequencing data of the WGS can be found in "Genome Sequence Archive for Human" (https://ngdc.cncb.ac.cn/gsa-human, accession No. HRA004900). The metagenomic sequencing data can be found in "Sequence Read Archive" (https://www.ncbi.nlm.nih.gov/sra, accession No. PRJNA986283 and PRJNA1011937). The GWAS data, characteristics of the cohorts, serum SCFA levels, and relative abundance of GM and KEGG modules have been deposited in the Figshare database (https://figshare.com, doi: 10.6084/m9.figshare.23267351). All the data are subject to open access.

Research involving human participants, their data, or biological material

Policy information about studies with human participants or human data. See also policy information about sex, gender (identity/presentation), and sexual orientation and race, ethnicity and racism.

Reporting on sex and gender	All of the subjects in this study are female.
Reporting on race, ethnicity, or other socially relevant groupings	N/A
Population characteristics	517 independent unrelated peri-/post-menopausal Chinese women were recruited for discovery. The mean (standard deviation) of age was 52.85 ± 2.92 years. 54.5% of the subjects had normal bone mineral density (BMD), 38.5% osteopenia and 7% osteoporosis. These subjects did not use antibiotics, oestrogens, anticonvulsant or proton pump inhibitor medications in the past three months; and they did not receive a treatment (e.g., bisphosphonates) that would be an apparent non-genetic factor underlying the variation of BMD. 59 US white subjects recruited for another study were used for partial validation. Individuals who had pathological conditions that may influence BMD (e.g., a bilateral oophorectomy, chronic renal failure, liver failure, lung diseases, gastrointestinal diseases, and inherited bone diseases), or may influence gut microbiota (e.g., taking antibiotics, having gastroenteritis, major surgery involving hospitalization, and inter-continental travel in the past three months) were excluded. The mean (standard deviation) of age was 66.98 ± 5.65 years.
Recruitment	All the subjects were randomly recruited. We used stringent inclusion and exclusion criteria to ensure that subjects were relatively homogeneous for age, ovarian function and living environment (details about the inclusion and exclusion criteria are shown in the manuscript and supplementary information) to minimize potential confounding factors and enhance the statistical power of our study. Therefore, there are no self-selection bias or other biases that may impact results.
Ethics oversight	This study was reviewed and approved by the Third Affiliated Hospital of Southern Medical University (Guangzhou City, China).

Note that full information on the approval of the study protocol must also be provided in the manuscript.

Field-specific reporting

Please select the one below that is the best fit for your research. If you are not sure, read the appropriate sections before making your selection.

☒ Life sciences ☐ Behavioural & social sciences ☐ Ecological, evolutionary & environmental sciences

For a reference copy of the document with all sections, see nature.com/documents/nr-reporting-summary-flat.pdf

Life sciences study design

All studies must disclose on these points even when the disclosure is negative.

Sample size	We totally recruited 517 Chinese subjects for discovery, plus 59 US white subjects for validation. We determined this sample size based on other reported research. This is currently the largest shotgun metagenomics study directly testing associations between gut microbiota and human bone mineral density.<br>Meanwhile, we determined the sample size of mice (n = 12/group) by referring to previous research (Arifin WN, Zahiruddin WM. Sample Size Calculation in Animal Studies Using Resource Equation Approach. Malays J Med Sci. 2017 Oct;24(5):101-105).
Data exclusions	Briefly, exclusion criteria included the use of antibiotics, oestrogens, or anticonvulsant medications which may affect gut microbiota composition and/or bone metabolism in the past three months, as well as other diseases that could lead to secondary osteoporosis. Additionally, we excluded rare gut species (relative abundance < 0.10%) because rare species typically contribute significantly less to functional diversity than non-rare species due to their lower abundances; meanwhile, the rare species are generally more difficult to cultivate, which might make our subsequent experimental validation in vivo difficult.

Replication	For the human study, we replicated the association between gut microbiota and bone mineral density in an independent cohort of US white females. For the in vivo studies in mice, we re-perforemd the experiments by another batch of mice (from n = 9/group to n = 12/group). For the in vitro studies in cells, we repeated each of the experiments for three or six times, respectively.
Randomization	We randomly recruited 517 independent unrelated peri-/post-menopausal Chinese women as subjects for discovery, plus 59 randomly recruited US white subjects for validation. We treated bone mineral density (BMD) as a continuous variable and then explored the associations among gut microbiota, short chain fatty acids and BMD by using several association analysis methods; therefore, the human participants are not need to be allocated into different experimental groups. All the mice were separated randomly into nine intended treatment groups (n = 12/group). The phenotypes of these subjects were blinded during all the subsequent experiments.
Blinding	The phenotypes of these subjects were blinded during all the subsequent experiments.

Reporting for specific materials, systems and methods

We require information from authors about some types of materials, experimental systems and methods used in many studies. Here, indicate whether each material, system or method listed is relevant to your study. If you are not sure if a list item applies to your research, read the appropriate section before selecting a response.

Materials & experimental systems

n/a	Involved in the study	
☐	☒	Antibodies
☐	☒	Eukaryotic cell lines
☒	☐	Palaeontology and archaeology
☐	☒	Animals and other organisms
☒	☐	Clinical data
☒	☐	Dual use research of concern
☒	☐	Plants

Methods

n/a	Involved in the study	
☒	☐	ChIP-seq
☒	☐	Flow cytometry
☒	☐	MRI-based neuroimaging

Antibodies

Antibodies used	(1) p-RELA (Absin, Shanghai, China), rabbit polyclonal antibody, ABS143574-50UL, Lot C10A023;<br>(2) p-CHUK/IKBKB (Cell Signaling Technology, Danvers, MA, USA), Rabbit, 2697T, Lot 21;<br>(3) p-NFKBIA (Cell Signaling Technology, Danvers, MA, USA), Rabbit, 2859T, Lot 18;<br>(4) GAPDH (Cell Signaling Technology, Danvers, MA, USA), Mouse, 97166T, Lot 6.<br>The dilutions used for each antibody followed the manuals/protocols of the manufacturer and were mentioned in the methods section of the manuscript.
Validation	The validation of each primary antibody is shown on the manufacturers' websites (www.absin.cn; www.cellsignal.com).

Eukaryotic cell lines

Policy information about cell lines and Sex and Gender in Research

Cell line source(s)	MC3T3-E1, RAW264.7 (Beina Chuanglian Biotechnology Institute, Beijing, China)
Authentication	Short tandem repeat (STR) profiling, implemented by Biowing applied biotechnology Co. Ltd (Shanghai City, China; www.biowing.com.cn), was used for cell line authentication.
Mycoplasma contamination	All cell lines tested negative for mycoplasma contamination.
Commonly misidentified lines (See ICLAC register)	No commonly misidentified cell lines were used.

Animals and other research organisms

Policy information about studies involving animals; ARRIVE guidelines recommended for reporting animal research, and Sex and Gender in Research

Laboratory animals	Seven-week-old female C57BL/6J mice (specific pathogen free grade) were purchased from the Guangdong Medical Laboratory Animal Center (Foshan City, China) and raised there. Five mice were kept in one box. The ambient temperature and humidity are 20-26℃ and 40%-70%, respectively. The dark/light cycle is 12 h: 12 h.
Wild animals	The study did not involve wild animals.

Reporting on sex	All of the mice in this study are female.
Field-collected samples	The study did not involve samples collected from the field.
Ethics oversight	All the procedures involving mice were approved by the Ethical Committee of Experimental Animal Science and Technology of Guangdong Medical Laboratory Animal Center (Foshan City, China).

Note that full information on the approval of the study protocol must also be provided in the manuscript.

Plants

Seed stocks	N/A
Novel plant genotypes	N/A
Authentication	N/A
